# Prognostic Value of Pretreatment Controlling Nutritional Status Score for Patients With Pancreatic Cancer: A Meta-Analysis

**DOI:** 10.3389/fonc.2021.770894

**Published:** 2022-01-20

**Authors:** Xiaofeng Ma, Weihua Zou, Yu Sun

**Affiliations:** Clinical Laboratory, Huzhou Central Hospital, Affiliated Central Hospital of Huzhou University, Huzhou, China

**Keywords:** CONUT score, pancreatic cancer, meta-analysis, prognostic factors, evidence-based medicine

## Abstract

**Background:**

Previous studies have explored the prognostic value of the pretreatment Controlling Nutritional Status (CONUT) score of patients with pancreatic cancer. However, the results of those studies were inconsistent. We used meta-analysis to investigate the impact of the CONUT score on the prognosis for patients with pancreatic cancer.

**Methods:**

We thoroughly searched the PubMed, Web of Science, Embase, and Cochrane Library databases for relevant articles from inception to November 19, 2021. Combined hazard ratios (HRs) and 95% confidence intervals (95% CIs) were used to estimate the prognostic value of the CONUT score with respect to survival duration. The pooled odds ratios (ORs) and 95% CIs were used to estimate the correlation between the CONUT score and clinical characteristics.

**Results:**

The database search found seven studies with 2,294 patients for inclusion in this meta-analysis. A high CONUT score was significantly associated with poor overall survival (OS) (HR = 1.56, 95% CI = 1.13–2.16, *p* = 0.007), but not with recurrence-free survival (RFS) (HR = 1.47, 95% CI = 0.97–2.23, *p* = 0.072) of patients with pancreatic cancer. Moreover, there was a significant association between an elevated CONUT score and male patients (OR = 1.34, 95% CI = 1.03–1.75, *p* = 0.029). However, there was no significant association between the CONUT score and the clinical stage (OR = 1.11, 95% CI = 0.46–2.71, *p* = 0.576), lymph node metastasis (OR = 0.73, 95% CI = 0.39–1.36, *p* = 0.323), lymphatic vessel invasion (OR = 0.84, 95% CI = 0.55–1.28, *p* = 0.411), invasion of the portal vein system (OR = 1.04, 95% CI = 0.51–2.13, *p* = 0.915), and nerve plexus invasion (OR = 1.22, 95% CI = 0.83–1.80, *p* = 0.318) in patients with pancreatic cancer.

**Conclusions:**

The results of our meta-analysis indicate that a high CONUT score predicts a poor OS in patients with pancreatic cancer. The CONUT score may be an effective prognostic factor in pancreatic cancer in clinical practice.

## Introduction

Pancreatic cancer is the seventh most deadly cancer worldwide (1). In 2018, there were 458,918 new cases of pancreatic cancer and 432,242 deaths (1). Pancreatic cancer has a high mortality rate. Although the diagnosis and treatment of pancreatic cancer have greatly advanced over the last several decades, its prognosis remains dismal (2). The 5-year survival rate for all stages of pancreatic cancer is 4.2% (3). The prognosis for patients with metastatic disease is poor, with a 5-year survival rate of 17.4% for patients who undergo surgical resection and only 0.9% for patients who do not undergo resection (3). Therefore, identification of novel and cost-effective biomarkers that can predict the prognosis of patients with pancreatic cancer and provide guidance for individualized treatment is urgently needed.

Increasing evidence has shown that the nutritional status and inflammatory status of the patient play pivotal roles in the development and progression of cancer (4). Many nutritional assessment biomarkers, including the C-reactive protein-to-albumin ratio (5), prognostic nutritional index (6), albumin-to-globulin ratio (7), modified Glasgow prognostic score (8), and the Controlling Nutritional Status (CONUT) score, have been used to predict the prognosis of patients with pancreatic cancer (9, 10). In 2005, Ignacio de Ulíbarri first proposed CONUT and used it to evaluate the nutritional status of patients (11). The calculation of the CONUT score is based on serum albumin, total lymphocyte count, and total cholesterol level (11). The CONUT scoring system is shown in **Table 1**; the score ranges from 0 to 12. The nutritional status of patients with CONUT scores of 0–1, 2–4, 5–8, and 9–12 is normal, light, moderate, and severe, respectively. The higher the CONUT score, the worse the nutritional status. Many studies have investigated the prognostic role of the CONUT score for patients with pancreatic cancer; however, the results of these studies were inconsistent (9, 10, 12–16). Therefore, we performed this meta-analysis to investigate the prognostic and clinicopathological significance of the CONUT score for patients with pancreatic cancer.

**Table 1:** The CONUT scoring system.

Parameters	Degree			
	Normal	Light	Moderate	Severe
Serum albumin (g/dL)	3.5–4.5	3.0–3.49	2.5–2.99	<2.50
Score	0	2	4	6
Total serum cholesterol (mg/dL)	≥180	140–180	100–139	<100
Score	0	1	2	3
Total lymphocyte count (/mm^3^)	≥1600	1200–1599	800–1199	<800
Score	0	1	2	3

CONUT, controlling nutritional status.

## Materials and Methods

### Literature Search

This meta-analysis was performed in accordance with the reporting guidelines of the Preferred Reporting Items for Systematic Reviews and Meta-Analyses (PRISMA) (17). We thoroughly searched the PubMed, Web of Science, Embase, and Cochrane Library databases for relevant articles from inception to November 19, 2021, using the following search items: “Controlling Nutritional Status”, “CONUT”, “pancreatic cancer”, “pancreatic carcinoma”, and “pancreatic neoplasms”. All searches were performed using a combination of MeSH terms and free-text words. The publication language was limited to English. References within the identified articles were manually examined to identify other potentially eligible studies.

### Inclusion and Exclusion Criteria

The inclusion criteria for a study were as follows: (1) patients were diagnosed with pancreatic cancer histologically; (2) the study reported the association between the pretreatment CONUT score and all survival outcomes, including but not limited to overall survival (OS), disease-free survival (DFS), and recurrence-free survival (RFS); (3) the hazard ratios (HRs) and 95% confidence intervals (95% CIs) were reported in the text or available according to the provided data; (4) a cutoff CONUT score was identified; and (5) the published study was in English. The exclusion criteria were as follows: (1) letters, reviews, comments, case reports, and meeting abstracts; (2) the study did not provide HRs and 95% CIs for analysis; (3) the study was a duplicate; and (4) the study was not on humans.

### Data Extraction and Quality Assessment

Two investigators (XM and WZ) independently assessed the eligible studies, and all disagreements were resolved by discussion with a third investigator (YS). The following information was extracted from each included study: first author’s name, year of publication, sample size, age, histological type, study design, tumor stage, treatment methods, cutoff CONUT score, study period, survival outcomes, survival analysis methods, and HRs and 95% CIs. The HRs and 95% CIs from multivariate analysis (MVA) were extracted, if provided; otherwise, the HRs and 95% CIs from univariate analysis (UVA) were used. MVA considers confounding factors and is more precise than UVA. Two investigators (XM and YS) independently assessed the quality of the eligible studies using the Newcastle–Ottawa Scale (NOS) (18). The NOS evaluates the methodological quality of a study with respect to patient selection, comparability of the study groups, and outcome assessment. The maximum NOS score is 9, so a study with a NOS score of ≥6 was considered high quality.

### Statistical Analysis

The combination of HRs and 95% CIs was used to estimate the prognostic value of the CONUT score for predicting survival duration. Heterogeneity among the studies in this meta-analysis was evaluated using Cochran’s Q test and the I^2^ statistical methods. If I^2^ > 50% or *p* < 0.10, indicating significant heterogeneity among the studies, then a random-effect model was used; otherwise, a fixed-effect model was used. Subgroup analysis, stratified by various factors, was performed to investigate the source of heterogeneity and for further investigations. The pooled odds ratios (ORs) and 95% CIs were used to estimate the correlation between the CONUT score and the clinical characteristics of pancreatic cancer. Potential publication bias was evaluated using Begg’s test with funnel plots and Egger’s test. Statistical significance was set at *p* < 0.05. Stata software ver. 12.0 (Stata Corporation, College Station, TX, USA) was used for all statistical analyses.

### Ethical Statement

Ethical approval and patient consent were not required because all analyses were based on previously published studies.

## Results

### Characteristics of Included Articles

**Figure 1:**
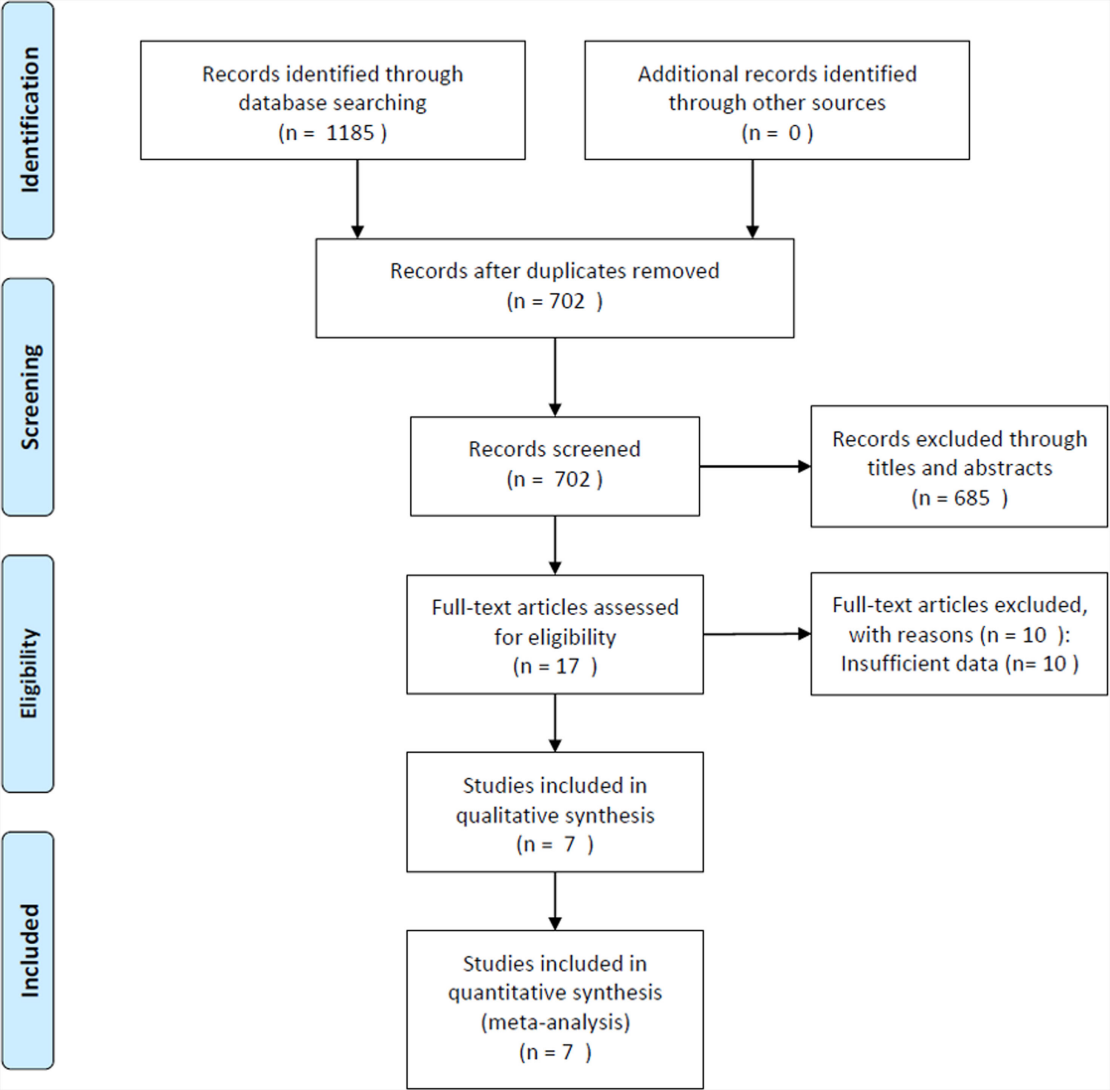
Flowchart of study selection for inclusion in the meta-analysis.

Seven studies with 2,294 patients (9, 10, 12–16) were included in this meta-analysis. The procedure used to select the included articles is presented in the flow diagram in **Figure 1**. The baseline characteristics of the seven included studies are presented in **Table 2**. Four studies were conducted in Japan (9, 10, 13, 16), and three were performed in China (12, 14, 15). The sample size ranged from 72 to 589, with a median of 307. Six studies were retrospective (9, 10, 12–14, 16), and one was prospective (15). All studies included patients with pancreatic ductal adenocarcinoma (PDAC). Five studies (10, 12, 14–16) recruited patients who underwent surgery, one study enrolled patients who received chemotherapy (9), and one study enrolled patients who underwent surgery and chemotherapy (13). Four studies (9, 12, 14, 16) used a cutoff CONUT score of ≥3, two (10, 13) used a cutoff CONUT score of ≥4, and one (15) used a cutoff CONUT score of ≥2. All seven studies (9, 10, 12–16) reported the prognostic role of the CONUT score for predicting OS, and five studies (9, 10, 13, 14, 16) presented the prognostic impact of the CONUT score on the RFS. The NOS scores of the seven studies ranged from 7 to 9, indicating that all included studies were of high quality.

**Table 2:** Baseline characteristics of included in this meta-analysis.

Study	Year	Country	Sample size	Sex (M/F)	Age	Histological type	Study design	Tumor stage	Treatment	Cutoff value	Study period	Survival endpoint	Survival analysis	NOS
Asama, H. (9)	2018	Japan	72	40/32	63 (42–85)	PDAC	Retrospective	III–IV	Chemotherapy	≥3	2006–2016	OS, RFS	MVA	8
Kato, Y. (10)	2018	Japan	344	201/137	64.8	PDAC	Retrospective	I–IV	Surgery	≥4	2002–2016	OS, RFS	MVA	9
Mao, Y. S. (12)	2020	China	306	186/120	62	PDAC	Retrospective	I–III	Surgery	≥3	2012–2014	OS	UVA	7
Terasaki, F. (13)	2021	Japan	307	182/125	NA	PDAC	Retrospective	I–IV	Surgery+chemotherapy	≥4	2007–2015	OS, RFS	MVA	7
Wang, A. (14)	2020	China	294	163/131	55.5 (29–78)	PDAC	Retrospective	I–III	Surgery	≥3	2012–2019	OS, RFS	MVA	8
Dang, C. (15)	2021	China	382	157/161	57.5 (28–78)	PDAC	Prospective	I–IV	Surgery	≥2	2014–2018	OS	MVA	9
Itoh, S. (16)	2021	Japan	589	326/263	71 (63–77)	PDAC	Retrospective	I–III	Surgery	≥3	2004–2016	OS, RFS	MVA	8

M, male; F, female; PDAC, pancreatic ductal adenocarcinoma; OS, overall survival; RFS, recurrence-free survival; MVA, multivariate analysis; UVA, univariate analysis; NA, not available; NOS, Newcastle–Ottawa Scale.

### Prognostic Value of CONUT Score for Predicting OS in Pancreatic Cancer

**Figure 2:**
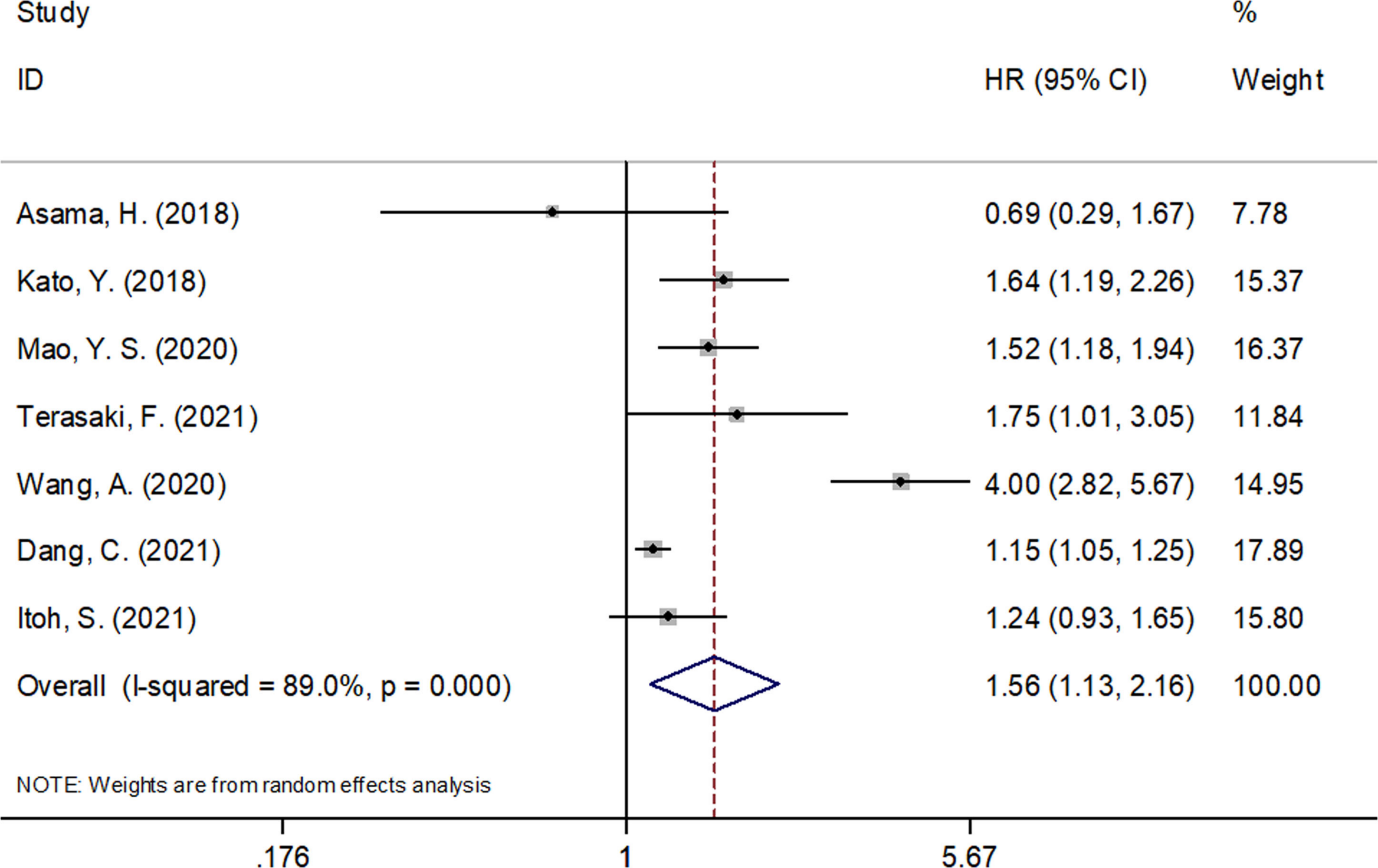
Forest plots of CONUT score in predicting OS in pancreatic cancer.

The seven included studies of this meta-analysis (9, 10, 12–16) reported the prognostic efficiency of the CONUT score in predicting OS. Because significant heterogeneity (I^2^ = 89.0%, *p* < 0.001) was detected, a random-effect model was used. As shown in **Figure 2** and **Table 3**, the pooled HR and 95% CI were HR = 1.56, 95% CI = 1.13–2.16, *p* = 0.007, suggesting that a high CONUT score was significantly associated with a poor OS for patients with pancreatic cancer. Subgroup analysis found that an elevated CONUT score (HR = 1.67, 95% CI = 1.15–2.41, *p* = 0.007) predicted a poor OS for patients undergoing surgery. Additionally, a cutoff CONUT score of ≥4 (HR = 1.67, 95% CI = 1.26–2.20, *p* < 0.001) predicted a poor OS for patients with pancreatic cancer (**Table 3**).

**Table 3:** The prognostic value of COUNT score for OS and RFS in pancreatic cancer and subgroup analysis.

Variables	No. of studies	No. of patients	HR (95% CI)	p	Heterogeneity		Effects model
					I^2^ (%) Ph		
Overall survival							
Total	7	2,294	1.56 (1.13–2.16)	0.007	89.0	<0.001	Random
Country							
Japan	4	1,312	1.39 (1.15–1.69)	0.001	37.2	0.189	Fixed
China	3	982	1.87 (1.01–3.47)	0.048	95.9	<0.001	Random
Sample size							
<330	3	685	1.74 (0.75–4.02)	0.198	92.2	<0.001	Random
≥330	4	1,609	1.31 (1.08–1.60)	0.007	54.2	0.088	Random
Treatment							
Surgery	5	1,915	1.67 (1.15–2.41)	0.007	92.2	<0.001	Random
Surgery + chemotherapy/chemotherapy	2	379	1.17 (0.47–2.89)	0.731	67.8	0.078	Random
Cut-off value							
≥2	1	382	1.15 (1.05–1.25)	0.002	–	–	–
≥3	4	1,261	1.61 (0.89–2.91)	0.112	90.8	<0.001	Random
≥4	2	651	1.67 (1.26–2.20)	<0.001	0	0.842	Fixed
Survival analysis							
MVA	6	1,988	1.56 (1.04–2.34)	0.030	90.5	<0.001	Random
UVA	1	306	1.52 (1.18–1.94)	0.001	–	–	–
Recurrence-free survival							
Total	5	1,606	1.47 (0.97–2.23)	0.072	85.6	<0.001	Random
Country							
Japan	4	1,312	1.23 (0.95–1.59)	0.121	50.7	0.107	Random
China	1	294	2.93 (2.10–4.09)	<0.001	–	–	–
Sample size							
<330	3	673	1.83 (1.03–3.25)	0.039	78.6	0.009	Random
≥330	2	933	1.11 (0.92–1.35)	0.267	0	0.971	Fixed
Treatment							
Surgery	3	1,227	1.53 (0.85–2.73)	0.155	91.8	<0.001	Random
Surgery + chemotherapy/chemotherapy	2	379	1.37 (0.65–2.85)	0.406	67.9	0.078	Random
Cutoff value							
≥3	3	955	1.47 (0.69–3.13)	0.318	90.7	<0.001	Random
≥4	2	651	1.41 (0.85–2.34)	0.187	78.3	0.032	Random

COUNT, controlling nutritional status; OS, overall survival; RFS, recurrence-free survival; MVA, multivariate analysis; UVA, univariate analysis.

### Prognostic Value of CONUT Score for Predicting RFS in Pancreatic Cancer

**Figure 3:**
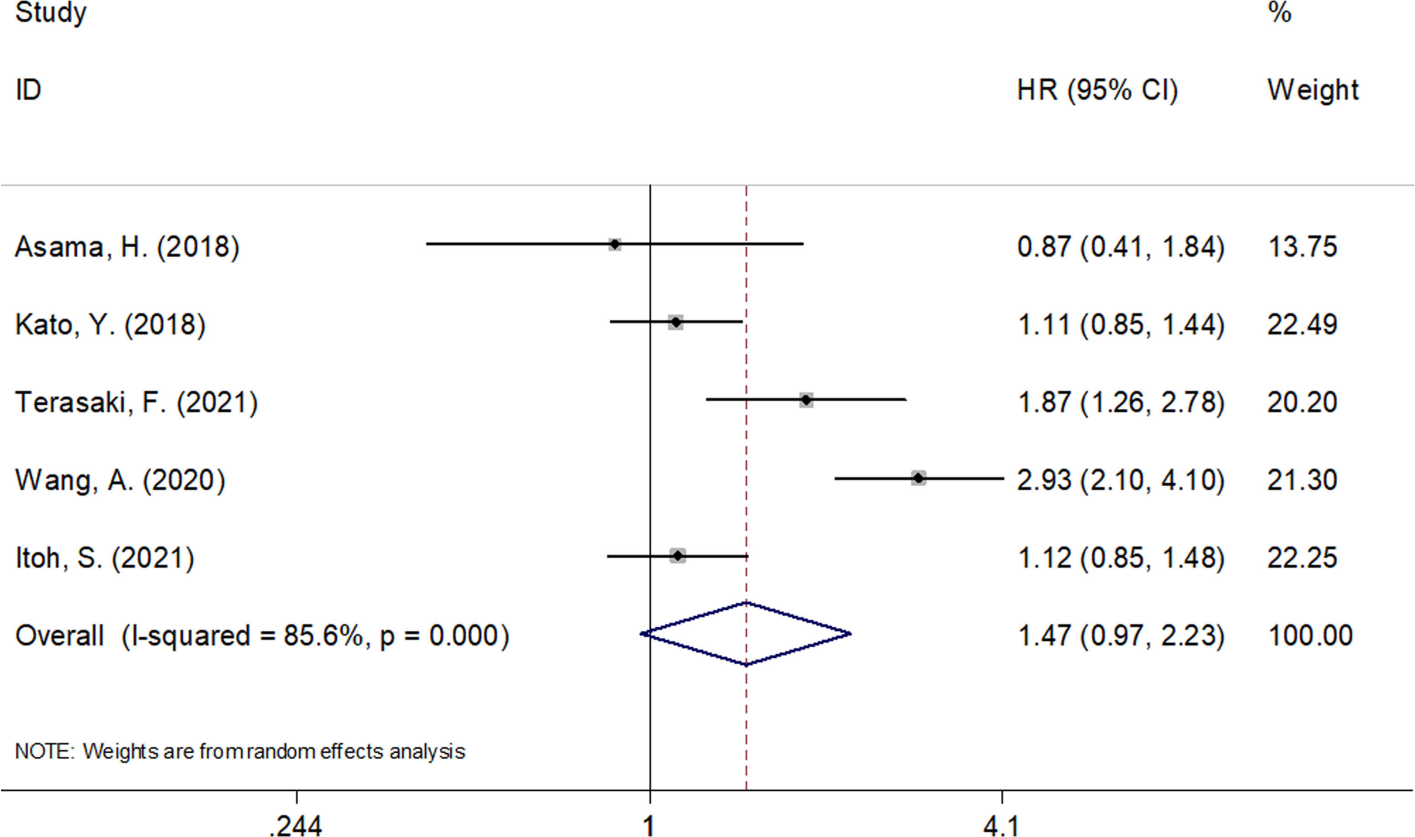
Forest plots of CONUT score in predicting RFS in pancreatic cancer.

Five studies consisting of 1,606 patients (9, 10, 13, 14, 16) investigated the prognostic value of the CONUT score in predicting RFS. As shown in **Figure 3** and **Table 3**, an elevated CONUT score was not significantly associated with the RFS of patients with pancreatic cancer (HR = 1.47, 95% CI = 0.97–2.23, *p* = 0.072). A random-effect model was used because of significant heterogeneity (I^2^ = 85.6%, *p* < 0.001). Subgroup analysis showed that a high CONUT score predicted a poor RFS for Chinese patients with pancreatic cancer (HR = 2.93, 95% CI = 2.10–4.09, *p* < 0.001) and in studies with sample size <330 (HR = 1.83, 95% CI = 1.03–3.25, *p* = 0.039) (**Table 3**). However, the CONUT score was not significantly associated with RFS irrespective of treatment or the cutoff CONUT value.

### Association Between CONUT Score and Patient Characteristics

We investigated the correlation between the CONUT score and the clinicopathological characteristics of patients with pancreatic cancer using the data from five studies with 1,633 patients (10, 12–15). As shown in **Table 4**, a high CONUT score was significantly associated with male patients (OR = 1.34, 95% CI = 1.03–1.75, *p* = 0.029). However, the association between the CONUT score and the clinical stage (OR = 1.11, 95% CI = 0.46–2.71, p = 0.576), lymph node metastasis (OR = 0.73, 95% CI = 0.39–1.36, p = 0.323), lymphatic vessel invasion (OR = 0.84, 95% CI = 0.55–1.28, p = 0.411), invasion of the portal vein system (OR = 1.04, 95% CI = 0.51–2.13, p = 0.915), and nerve plexus invasion (OR = 1.22, 95% CI = 0.83–1.80, p = 0.318) in pancreatic cancer was not significant (**Table 4**).

**Table 4:** The association between COUNT score and clinicopathological features in patients with pancreatic cancer.

Factors	No. of studies	No. of patients	OR (95% CI)	p	Heterogeneity		Effects model
					I^2^ (%) Ph		
Sex (male vs. female)	4	1,289	1.34 (1.03–1.75)	0.029	29.6	0.235	Fixed
Clinical stage (III–IV vs. I–II)	4	1,289	1.11 (0.46–2.71)	0.576	85.7	<0.001	Random
Lymph node metastasis (positive vs. negative)	5	1,633	0.73 (0.39–1.36)	0.323	80.8	<0.001	Random
Lymphatic vessel invasion (positive vs. negative)	2	638	0.84 (0.55–1.28)	0.411	0	0.957	Fixed
Invasion of portal vein system (positive vs. negative)	2	638	1.04 (0.51–2.13)	0.915	73.6	0.052	Random
Nerve plexus invasion (positive vs. negative)	2	638	1.22 (0.83–1.80)	0.318	0	0.663	Fixed

COUNT, controlling nutritional status.

### Publication Bias

**Figure 4:**
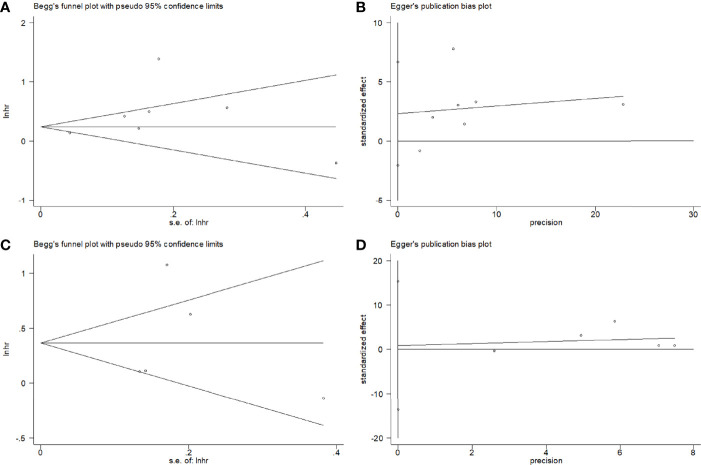
Publication bias test by Begg’s funnel plot and Egger’s test. **(A)** Begg’s test for OS, *p* = 0.764; **(B)** Egger’s test for OS, *p* = 0.717; **(C)** Begg’s test for RFS, p = 0.463; **(D)** Egger’s test for RFS, *p* = 0.792.

Potential publication bias was evaluated using Begg’s test and Egger’s test. **Figure 4** shows there is no significant publication bias with respect to OS (Begg’s test: *p* = 0.764; Egger’s test: *p* = 0.717) and RFS (Begg’s test: *p* = 0.463, Egger’s test: *p* = 0.792).

## Discussion

In recent years, the prognostic value of inflammatory and nutritional indices of patients with cancer has been extensively investigated (19–21). Studies have investigated the prognostic value of the CONUT score for patients with pancreatic cancer, but the results were inconsistent. In our meta-analysis, we combined data from seven studies that comprised 2,294 patients. The pooled data demonstrated that a high CONUT score was a significant prognostic biomarker for predicting the OS but not the RFS of patients with pancreatic cancer. Additionally, an elevated CONUT score correlated with male patients with pancreatic cancer, which suggests that male patients with pancreatic cancer tend to have higher CONUT scores. Thus, a high CONUT score was a reliable prognostic factor for a poor OS for patients with pancreatic cancer and could be used to identify high-risk patients. To our knowledge, this meta-analysis was the first to investigate the prognostic value of the CONUT score of patients with pancreatic cancer.

The CONUT score was computed using serum albumin, total lymphocyte count, and total cholesterol values. Patients with low levels of these parameters have a low CONUT score. The biological mechanisms involved in the prognostic value of the CONUT score with respect to a poor OS for patients with pancreatic cancer are still not fully understood but could be explained by the following points. First, the serum albumin level is an important indicator of nutritional status and is regarded as an acute-phase protein that has a role in systemic inflammation (22). Pretreatment hypoalbuminemia correlates with poor survival of patients with cancer (23). Second, lymphocytes play a pivotal role in cell-mediated antitumor immune responses (24). Tumor-infiltrating lymphocytes are important components of antitumor activity and can induce cytotoxic cell death and inhibit tumor cell proliferation (25). Therefore, low lymphocyte counts can weaken immune responses and lead to poor survival for patients with cancer (26). Third, cholesterol is essential for the maintenance of cell membrane function, which is crucial for signal transduction. Decreased levels of cholesterol can affect the antitumor activity of immunocompetent cells (27). Therefore, a low CONUT score could represent a combination of a low serum albumin level, a low lymphocyte count, and a low total cholesterol level, which is reasonably associated with poor survival in patients with cancer.

Many recent meta-analysis studies also have investigated the prognostic role of the CONUT score with respect to solid tumors. In a meta-analysis that included 3,029 patients, Peng et al. showed that a high CONUT score positively correlated with poor prognoses in patients with non-small cell lung cancer (28). Another meta-analysis of six studies demonstrated that a high CONUT score correlated with a poor OS, cancer-specific survival, and DFS in patients with upper tract urothelial carcinoma or renal cell carcinoma undergoing nephrectomy (29). A meta-analysis by Takagi et al. showed that the preoperative CONUT score was an independent prognostic indicator of survival in patients with gastric cancer (30). Our meta-analysis investigated the prognostic efficiency of the CONUT score for patients with pancreatic cancer. We identified a positive association between the CONUT score and the OS of patients with pancreatic cancer, whereas its prognostic role with respect to the RFS was not significant, maybe because of the relatively short follow-up period for RFS analysis and the small sample size (only five studies were included in the RFS analysis). The prognostic value of the CONUT score for predicting the RFS in pancreatic cancer should be verified by future large-scale trials.

This meta-analysis has several limitations. First, the sample size was relatively small. Only seven studies with 2,294 patients were included in the analysis. The small sample size may have introduced a selection bias in this meta-analysis because when the sample size is small, the data of each individual could have a greater impact on the overall results than when the sample size is larger (31). Second, all included studies were conducted in Asian countries, namely, Japan and China. Although we thoroughly searched the literature, no studies of non-Asian patients met our inclusion criteria. Third, the included studies did not have the same cutoff CONUT scores; four studies used ≥3, two studies used ≥4, and one study used ≥2. The different cutoff CONUT scores may have caused the heterogeneity among the studies.

## Conclusions

In summary, our meta-analysis showed that a high CONUT score is significantly associated with a poor OS for patients with pancreatic cancer. Male patients with pancreatic cancer tend to have a higher CONUT score. The CONUT score may be an effective prognostic factor in pancreatic cancer in clinical practice.

## Data Availability Statement

All datasets generated for this study are included in the article/supplementary material. Further inquiries can be directed to the corresponding author.

## Author Contributions

XM and WZ collected, extracted, and performed the quality assessment and analyzed the data; YS conceived and designed this study and wrote the paper. All authors reviewed the final manuscript. All authors contributed to the article and approved the submitted version.

## Conflict of Interest

The authors declare that the research was conducted in the absence of any commercial or financial relationships that could be construed as a potential conflict of interest.

## Publisher’s Note

All claims expressed in this article are solely those of the authors and do not necessarily represent those of their affiliated organizations, or those of the publisher, the editors and the reviewers. Any product that may be evaluated in this article, or claim that may be made by its manufacturer, is not guaranteed or endorsed by the publisher.
